# Leprosy in Nonimmigrant Canadian Man without Travel outside North America, 2014

**DOI:** 10.3201/eid2401.170547

**Published:** 2018-01

**Authors:** Paul E. Bonnar, Natalie P. Cunningham, Andrea K. Boggild, Noreen M. Walsh, Rahul Sharma, Ian R.C. Davis

**Affiliations:** Dalhousie University, Halifax, Nova Scotia, Canada (P.E. Bonnar, N.P. Cunningham, N.M. Walsh, I.R.C. Davis);; University of Toronto, Toronto, Ontario, Canada (A.K. Boggild);; Toronto General Hospital, Toronto (A.K. Boggild);; Public Health Ontario Laboratories, Toronto (A.K. Boggild);; National Hansen’s Disease Program, Baton Rouge, Louisiana, USA (R. Sharma)

**Keywords:** armadillos, microbiology, Canada, epidemiology, disease reservoirs, humans, leprosy, *Mycobacterium leprae*, classification, genetics, United States, Florida, bacteria, zoonoses, tuberculosis and other mycobacteria, North America

## Abstract

In Canada, Hansen disease (leprosy) is rare and not considered in diagnoses for nonimmigrant patients. We report *Mycobacterium leprae* infection in a Canadian man whose sole travel was to Florida, USA. The *M. leprae* isolate was identified as armadillo-associated genotype 3I-2-v1. Travelers to the southern United States should avoid contact with armadillos.

In 2014, a 69-year-old nonimmigrant man from Atlantic Canada who had a 10-month history of nonscaly, annular, polycyclic plaques over his trunk and extremities sought treatment. His lesions were red-brown and 4–5 cm in diameter (Technical Appendix Figure). He also had innumerable erythematous papules and plaques measuring 0.5–2 cm. These lesions were predominantly symmetric with poorly defined borders; skin was normal between lesions. His condition did not improve with a 30-day trial of triamcinolone cream and doxycycline. He had no sensation abnormalities, thickened peripheral nerves, motor neuropathy, or alopecia. His medical history included coronary artery disease, diabetes, dyslipidemia, and hypertension. His only travel consisted of yearly visits to Davenport, Florida, USA, for the past 8 years. He had no contact with animals, including armadillos, or with persons with similar lesions. The patient had always lived in Atlantic Canada and was a retired farmer who had never employed foreign workers.

An earlier punch biopsy, taken at another center and unavailable for review, was interpreted as suggestive of erythema annulare centrifugum. A second sample, obtained 10 months after disease onset, revealed a light perivascular lymphohistiocytic infiltrate, as seen in erythema annulare centrifugum. However, scant, interspersed foamy histiocytes consistent with Virchow-globi cells were noted. Fite staining confirmed their cytoplasmic content of abundant and focally clumped acid-fast bacilli. A slit skin examination before treatment had a bacteriologic index of 4+. A *Mycobacterium leprae*‒specific PCR targeting the *RLEP* gene (*1*) from skin tissue was positive, and 16S rRNA gene (GenBank accession no. NC_002677, region 1341144‒1342692 bp) sequencing also confirmed *M. leprae* infection. On the basis of the World Health Organization’s disease classification, the patient (having >5 skin lesions and positive skin slits) was given a diagnosis of multibacillary Hansen disease (HD). The Ridley-Jopling classification of borderline lepromatous leprosy was based on the absence of granulomas; numerous bacteria on staining; and symmetric, poorly defined skin lesions.

The patient was started on a 24-month course of ofloxacin, rifampin, and dapsone. Initially, his skin lesions improved, but after 10 months of treatment, he had nondermatomal sensory changes in his hands and erythema with induration of many preexisting lesions, indicating a reversal (type 1) reaction. He was promptly started on prednisone, and a repeat biopsy showed granuloma formation and a bacteriologic index of 1+ acid-fast bacilli, consistent with enhanced cell-mediated immunity. Two months after therapy, he had residual pigmentation without active inflammation and improving hand sensation.

To identify the probable source of infection, we genotyped the *M. leprae* from this patient using a single-nucleotide polymorphism and variable number tandem repeat (VNTR)‒based algorithm for identifying zoonotic *M. leprae* strains (*2**,**3*). We amplified and sequenced genomic regions spanning the markers (Technical Appendix Table). All the single-nucleotide polymorphisms and VNTR loci sequences were identical to the first zoonotic strain (3I-2-v1) of leprosy. The probability of an identical pattern in the random assortment of the 10 VNTRs alone is 1 in 10,000 (*2*), implying infection with a zoonotic strain of *M. leprae*.

HD, caused by *M. leprae*, is rare in Canada. In 2014, four cases were reported in Canada, and in 2015, a total of 178 new cases were reported in the United States (*4*,*5*). The diagnosis is challenging in low-incidence countries and often delayed for years (*6*). Neurologic dysfunction is a clue for the diagnosis but is often absent (*6*). Unrecognized source contact is common among HD patients (*7*), but exposure is doubtful to have occurred with this patient who had not traveled to an area considered endemic. In the southern United States, autochthonous cases of HD have occurred among native-born persons. In 2015, a total of 63 of the 96 cases reported from Texas, Louisiana, Arkansas, Mississippi, Alabama, Georgia, and Florida occurred among persons born in the United States who had never resided outside the country (*5*). These cases might be secondary to exposure to the 9-banded armadillo (*Dasypus novemcinctus*), which lives in the area (*2*). Most HD patients in Louisiana, Texas, and Florida who had not traveled outside the United States were infected with the *M. leprae* strain 3I-2-v1, which is found in most infected armadillos (*2*). Although direct contact with armadillo blood or flesh poses the highest risk, HD has been reported in persons without direct exposure (*8*). These patients might have had exposure to contaminated soil (*9*). Alternatively, other environmental reservoirs might be responsible, exemplified by the discovery of infected Eurasian red squirrels (*Sciurus vulgaris*) in the British Isles (*10*).

*M.* *leprae* genomic analysis strongly suggests that our patient acquired the infection with the armadillo-associated *M. leprae* strain during a trip to Florida. This case highlights the possibility of HD being acquired within North America without obvious exposure to known animal reservoirs. Travelers to the southern United States should be advised to avoid contact with armadillos.

Technical Appendix Genetic information on the *Mycobacterium leprae *strain isolated from patient and patient’s timeline of disease.
