# Supplementary material for: Leprosy in Nonimmigrant Canadian Man without Travel outside North America, 2014
Source: Emerg Infect Dis. 2018 Jan;24(1):165–6. doi: 10.3201/eid2401.170547 (PMC5749456; doi:10.3201/eid2401.170547)
Supplement: Technical Appendix — Genetic information on the Mycobacterium leprae strain isolated from patient and patient’s timeline of disease. [file 17-0547-Techapp-s1.pdf]

Article DOI: https://doi.org/10.3201/eid2401.170547

Leprosy in Nonimmigrant Canadian Man without Travel outside North America, 2014

Technical Appendix

Technical Appendix Table. *Mycobacterium leprae* genotype from nonimmigrant Canadian man with leprosy, 2014*

Genotyping markers used	SNP			VNTR									
	SNP	InDel	SNP	AC8A	AC8b	AC9	GGT5	GTA-9	6–7	12–5	21–3	23–3	27–5
Genome position	7614	17915	1527056	1531185–1531200	2211035–2211050	1452573–1452590	2567249–2567266	2583814–2583840	1816851–1816892	1381663–1381722	73074–7 3143	2945487–2945555	343053–343104
Standard strain TN	C	2	G	8	8	9	5	9	7	5	3	3	5
Zoonotic strain 3I-2-v1	T	1	C	10	7	8	4	10	7	5	2	2	4
Patient isolate 14–193	T	1	C	10	7	8	4	10	7	5	2	2	4

*We sequenced the *M. leprae* genome of strain TN (GenBank accession no. NC_002677.1) at positions 7614 and 17915, and a single copy of the 5-base repetitive sequence at 17915. T at SNP_7614 identified this sample as SNP type 3I. Sequencing of SNP 1527056 subtyped this sample as 3I-2, the only major SNP type reported from wild armadillos (*1*,*2*). SNP analysis established the geographic affiliation of this strain to North America. For further discrimination, we analyzed 10 VNTR loci as described earlier (*2*). PCR products were sequenced to determine the SNP and copy number of VNTR loci. SNP, single-nucleotide polymorphism; VNTR, variable number tandem repeat.

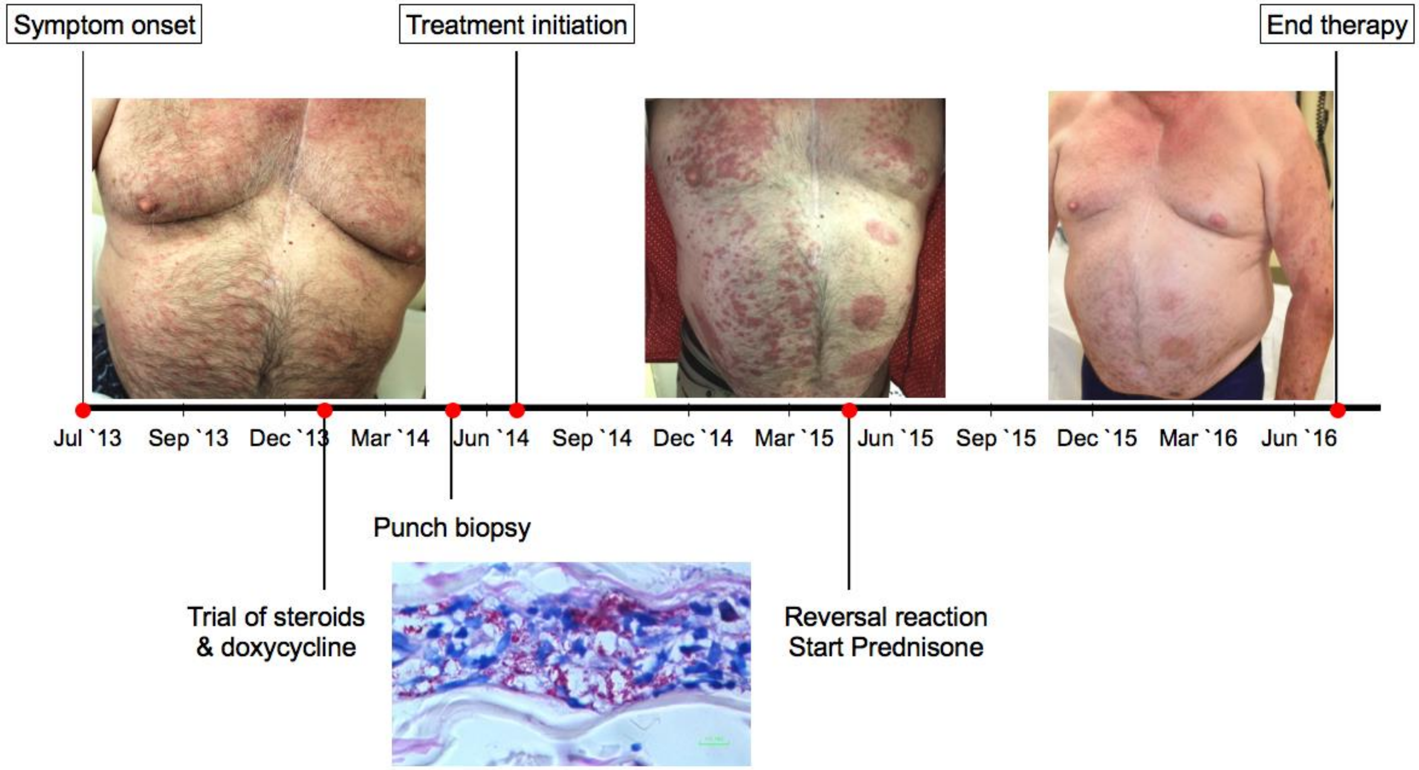

Technical Appendix Figure. Timeline of disease of nonimmigrant Canadian man with leprosy, 2014. Images show the progression of skin lesions before, during, and at the end of his 2-year therapy. The Fite-stained punch biopsy was positive for acid-fast bacilli (magenta stain), which were abundant within interspersed foamy histiocytes, consistent with Virchow-globi cells.

References

1. Truman RW, Singh P, Sharma R, Busso P, Rougemont J, Paniz-Mondolfi A, et al. Probable zoonotic leprosy in the southern United States. N Engl J Med. 2011;364:1626–33. PubMed http://dx.doi.org/10.1056/NEJMoa1010536

2. Sharma R, Singh P, Loughry WJ, Lockhart JM, Inman WB, Duthie MS, et al. Zoonotic leprosy in the southeastern United States. Emerg Infect Dis. 2015;21:2127–34. PubMed http://dx.doi.org/10.3201/eid2112.150501
